# Neonatal Presentation of 49,XXXXY (Fraccaro) Syndrome with Ventriculomegaly: Expanding the Early Neuroimaging Phenotype

**DOI:** 10.3390/pediatric18030076

**Published:** 2026-06-03

**Authors:** Gonca Vardar, Giray Girgin, Emel Kabakoglu Unsur, Gulcan Seymen

**Affiliations:** Department of Pediatrics, Acibadem Kartal Hospital, Istanbul 34873, Turkey

**Keywords:** 49,XXXXY, sex chromosome pentasomy, ventriculomegaly, colpocephaly, neonatal diagnosis

## Abstract

49,XXXXY syndrome (Fraccaro syndrome) is a rare sex chromosome pentasomy, historically considered a severe variant within the Klinefelter spectrum. It is characterized by intellectual disability, craniofacial dysmorphism, skeletal anomalies, hypogonadism, and congenital cardiac defects. Although neuroimaging abnormalities have increasingly been recognized in 49,XXXXY syndrome, neonatal diagnosis prompted primarily by ventriculomegaly remains rare. We report a neonate with prenatally detected ventriculomegaly in whom postnatal evaluation revealed cleft palate, congenital cardiac defects, bilateral cryptorchidism, and auditory dysfunction. Cranial ultrasonography and brain magnetic resonance imaging demonstrated bilateral ventriculomegaly with colpocephaly and a cavum vergae variant. Cytogenetic analysis confirmed the presence of a 49,XXXXY karyotype. This case highlights ventriculomegaly as a potential early diagnostic clue in 49,XXXXY syndrome and underscores the importance of chromosomal analysis in neonates presenting with structural brain abnormalities associated with multisystem anomalies. Early recognition is important for timely multidisciplinary surveillance and long-term endocrine follow-up.

## 1. Introduction

49,XXXXY syndrome was first described in 1960 and represents one of the rarest sex chromosome aneuploidies, with an estimated incidence of 1:85,000–100,000 male births [1]. Although historically considered a variant of Klinefelter syndrome, 49,XXXXY syndrome is increasingly recognized as a distinct clinical entity because of its characteristic and more severe multisystem manifestations [2]. Supporting this distinction, Peet et al. emphasized as early as 1998 that 49,XXXXY syndrome should be evaluated separately from classic Klinefelter syndrome because of its unique clinical features [3]. The classical clinical triad consists of intellectual disability, radioulnar synostosis, and hypogonadism. Affected individuals also frequently exhibit craniofacial dysmorphism, congenital heart defects, growth restriction, and genital anomalies [4].

Herein, we report a neonate with a karyotype of 49,XXXXY who presented with both prenatal and postnatal ventriculomegaly. The case is notable for the coexistence of cleft palate, ventriculomegaly, congenital cardiac defects, and cryptorchidism. Although several structural and developmental abnormalities have been described in association with 49,XXXXY syndrome, only rarely has ventriculomegaly been emphasized as the principal neonatal finding in a manner that prompts genetic evaluation. The present case highlights the potential role of prenatal and early postnatal neuroimaging findings in prompting genetic investigation and further expands the reported neuroradiological spectrum of the syndrome.

## 2. Case Presentation

A male infant was delivered at 37 weeks’ gestation via cesarean section to a 29-year-old primigravid mother. The parents were third-degree relatives. Prenatal ultrasonography demonstrated ventriculomegaly, although invasive prenatal genetic testing was declined. Birth weight was 2.2 kg (<3rd percentile), consistent with intrauterine growth restriction (IUGR). Apgar scores were 8, 9, and 9 at 1, 5, and 10 min, respectively. Shortly after birth, the neonate developed transient tachypnea, requiring short-term nasal continuous positive airway pressure support. Physical examination revealed hypertelorism, epicanthal folds, low-set ears, cleft palate, and a single transverse palmar crease on the right hand. Bilateral undescended testes were identified and localized within the inguinal canals by ultrasonography. No persistent Müllerian structures were detected (Figure 1).

Echocardiography demonstrated a 5 mm atrial septal defect (ASD), a small mid-muscular ventricular septal defect (VSD), and a mild patent ductus arteriosus (PDA) that closed spontaneously. Brainstem auditory evoked potentials (BAEP) revealed mild bilateral sensorineural hearing impairment. Ophthalmologic examination, including fundoscopy, was unremarkable. Radiographs of the upper and lower extremities showed no abnormalities, and no radiological evidence of radioulnar synostosis was identified. Complete blood count, liver function tests, and renal function tests were within normal limits. In addition, the neonatal period was not complicated by jaundice or hypoglycemic episodes.

Cranial ultrasonography confirmed bilateral ventriculomegaly. In the right hemisphere, the ventricular index measured 13.7 mm, the anterior horn width measured 6.5 mm, and the thalamo-occipital distance measured 16.4 mm. Corresponding measurements in the left hemisphere were 15.7 mm, 9.3 mm, and 21 mm, respectively, based on previously published neonatal ventricular reference values [5] (Figure 2).

Brain magnetic resonance imaging (MRI) revealed a thin-walled subependymal cystic lesion measuring 5.4 × 3.7 mm, with cerebrospinal fluid (CSF) signal intensity in the left caudothalamic notch. A cavum vergae variant with CSF signal intensity was observed at the midline between the lateral ventricles. Dilatation of both lateral ventricles was present; this was more pronounced on the left side and within the occipital horns, consistent with colpocephaly. Symmetrical intermediate signal intensity within the bilateral perirolandic white matter was considered compatible with the normal myelination process. The aqueduct of Sylvius was patent (Figure 3).

Overall, the patient demonstrated multisystem involvement, including ventriculomegaly with colpocephaly, congenital cardiac defects, cleft palate, bilateral cryptorchidism, and mild sensorineural hearing impairment.

Conventional karyotyping revealed a 49,XXXXY chromosomal constitution. The patient was discharged on day 7 of life in stable condition and enrolled in a multidisciplinary follow-up involving pediatric neurology and neurosurgery. The parents were informed that, although the child was genetically male, long-term follow-up by pediatric endocrinology would be required, particularly with regard to the potential need for hormone replacement therapy during puberty. Endocrinologic evaluation performed on postnatal day 25 demonstrated mildly elevated follicle-stimulating hormone (FSH) levels. Given the physiological variability of neonatal gonadotropin levels, these findings were interpreted cautiously according to postnatal age-specific reference ranges, as presented in Table 1.

Congenital infections within the TORCH spectrum, including toxoplasmosis, rubella, cytomegalovirus, herpes simplex virus, and syphilis, were systematically excluded through serological testing. Obstructive hydrocephalus as well as hemorrhagic and hypoxic–ischemic etiologies were considered unlikely based on the neuroimaging findings and an unremarkable perinatal history. In addition, metabolic causes of ventriculomegaly were excluded through metabolic screening, including serum ammonia, lactate, plasma amino acid analysis, and urine organic acid analysis.

## 3. Discussion

49,XXXXY syndrome is one of the rarest sex chromosome aneuploidies and is associated with prenatal growth restriction, cryptorchidism, and characteristic craniofacial abnormalities [6]. In the present case, the most notable finding was prenatal ventriculomegaly with persistence during the postnatal period, which prompted early genetic evaluation. Our patient presented with IUGR, hypertelorism, epicanthal folds, low-set ears, and bilateral undescended testes. Although the parents were third-degree relatives, 49,XXXXY syndrome is known to result from sporadic meiotic nondisjunction rather than inherited transmission, and no established association with consanguinity has been reported [2].

The classical clinical triad of 49,XXXXY syndrome consists of intellectual disability, radioulnar synostosis, and hypogonadism [3]. In addition, congenital cardiac malformations, reported in approximately 14% of cases, and cleft palate may also be observed. Patent ductus arteriosus is considered the most frequently reported congenital cardiac defect in this syndrome; in our patient, it was accompanied by both ventricular and atrial septal defects [4].

A comparison of previously reported neonatal or early-diagnosed cases of 49,XXXXY syndrome with the present case is summarized in Table 2.

Tabarki et al. reported white matter abnormalities, ventriculomegaly, corpus callosum hypoplasia, and cerebral volume loss on brain magnetic resonance imaging (MRI) in patients with 49,XXXXY syndrome [13]. Previous neuroimaging studies have shown that 49,XXXXY syndrome is associated with reduced brain volume and an increased frequency of structural brain abnormalities, including colpocephaly, plagiocephaly, and periventricular cysts. Colpocephaly is thought to result from underdevelopment of the adjacent cerebral white matter and may be more pronounced in the left occipital horn region, potentially contributing to the neurodevelopmental impairment observed in affected individuals [14]. Language and speech development are typically significantly delayed in these patients and are often characterized by dissociation between expressive and receptive language abilities [15].

In our patient, ventriculomegaly and colpocephaly were identified during the neonatal period, and the neuroimaging findings prompted early genetic evaluation, leading to the diagnosis of 49,XXXXY syndrome. In addition, brain MRI demonstrated a cavum vergae variant.

Previous reports have also documented auditory dysfunction and sensorineural hearing impairment in patients with 49,XXXXY syndrome [12]. Consistent with these observations, BAEP findings in our patient demonstrated mild bilateral sensorineural hearing impairment.

Several case reports have further highlighted the association between 49,XXXXY syndrome and genital anomalies. One report described a two-month-old infant presenting with a microphallus and a right undescended testis, whereas another neonatal case demonstrated a micropenis, perineal hypospadias, a bifid scrotum, and small palpable testes [11,15]. Similarly, bilateral undescended testes were identified in our patient. Hypogonadism remains one of the major defining clinical features of 49,XXXXY syndrome [9].

Hormonal profiles in patients with 49,XXXXY syndrome demonstrate considerable variability. During the prepubertal period, follicle-stimulating hormone (FSH), luteinising hormone (LH), and testosterone levels are generally within normal limits. In early puberty, elevations in LH and FSH are typically accompanied by only modest increases in testosterone levels [16]. Accordingly, evaluation for hypergonadotropic hypogonadism during puberty is recommended, and testosterone replacement therapy may be considered under endocrinological supervision to optimize clinical and developmental outcomes [15].

In our patient, mildly elevated FSH levels were detected during the neonatal period. However, the clinical significance of neonatal gonadotropin elevation in 49,XXXXY syndrome remains uncertain in the current literature, particularly given the physiological hormonal variability observed during early infancy. Therefore, long-term endocrinological follow-up was initiated in view of the established risk of future gonadal insufficiency and hypergonadotropic hypogonadism [16].

## 4. Conclusions

In summary, we report a neonatal case of 49,XXXXY syndrome in which ventriculomegaly served as an important early finding prompting genetic evaluation. In neonates presenting with ventriculomegaly in association with multisystem anomalies, including craniofacial, cardiac, or genital abnormalities, chromosomal analysis should be considered as part of the diagnostic evaluation. Early recognition and long-term multidisciplinary follow-up, including neurological, endocrinological, developmental, and audiological assessment, may contribute to improved clinical management and long-term outcomes in affected patients.

## Figures and Tables

**Figure 1:**
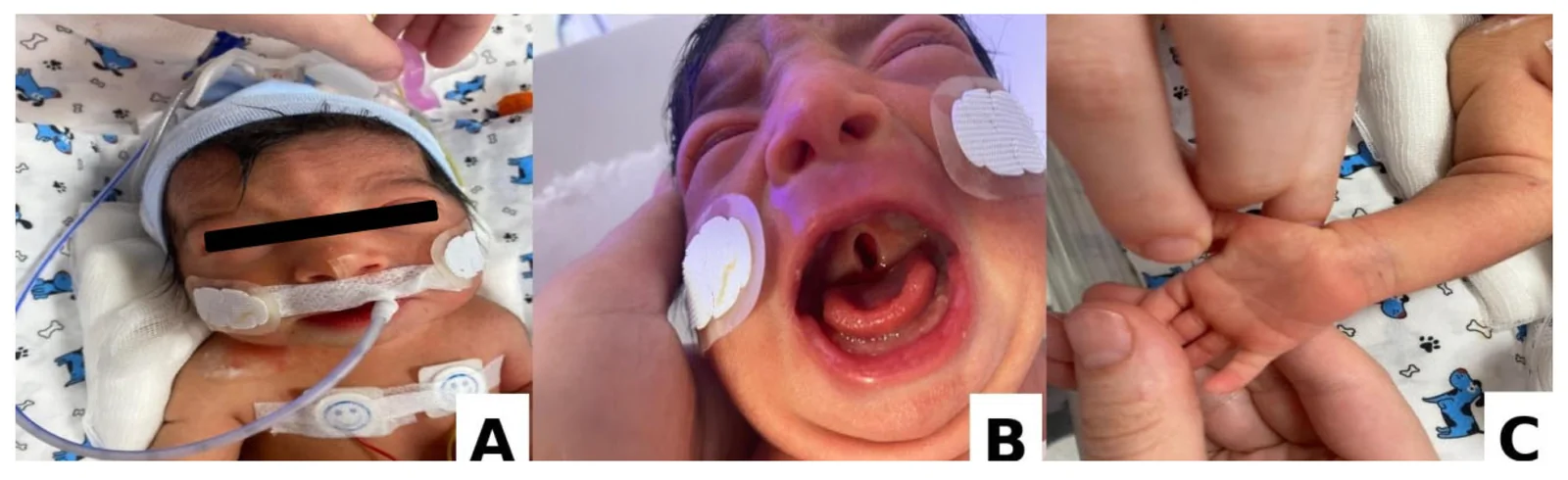
Clinical features of the patient with 49,XXXXY syndrome: (**A**) hypertelorism, (**B**) cleft palate, and (**C**) single transverse palmar crease.

**Figure 2:**
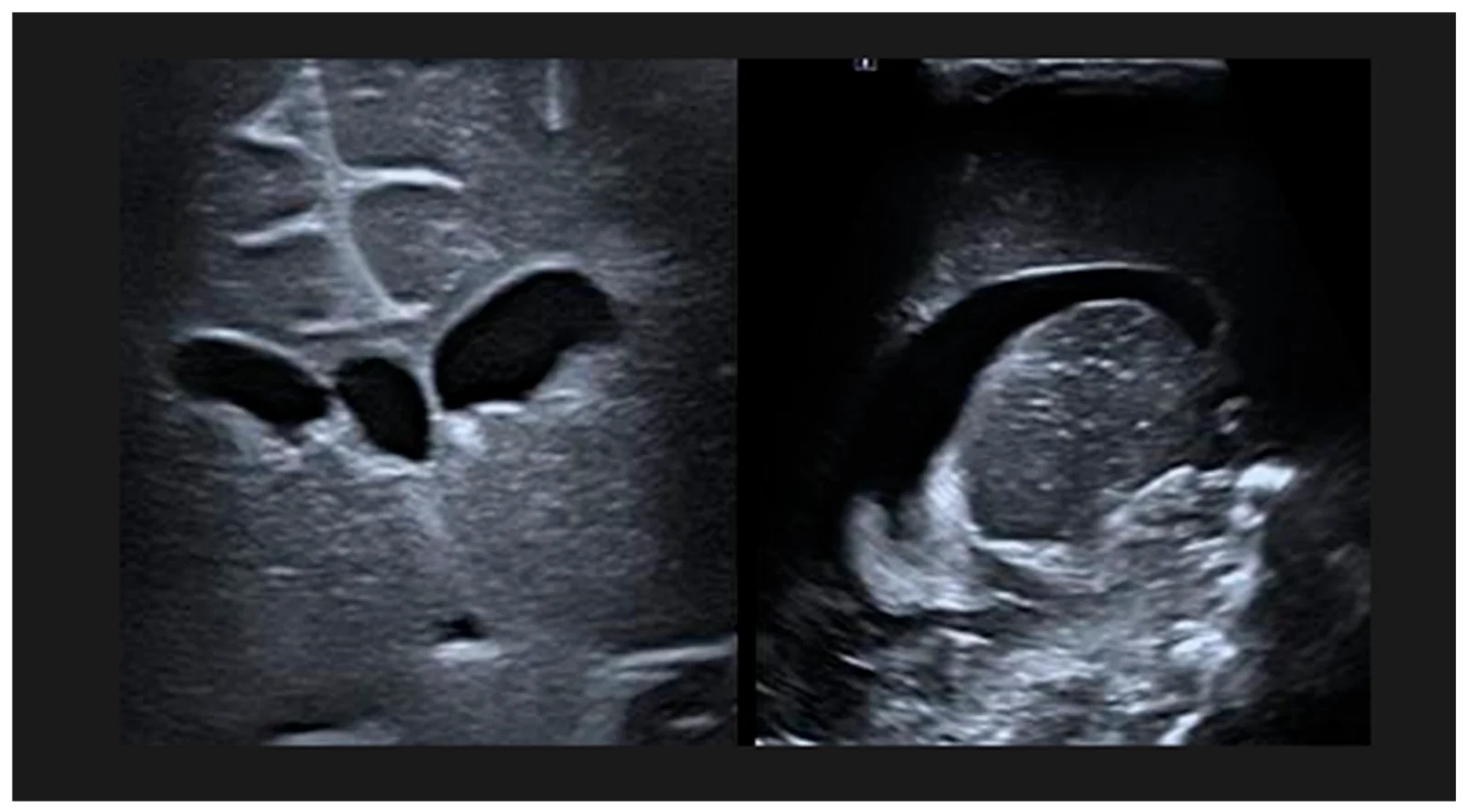
Cranial ultrasonography demonstrating bilateral ventriculomegaly, more pronounced in the left lateral ventricle.

**Figure 3:**
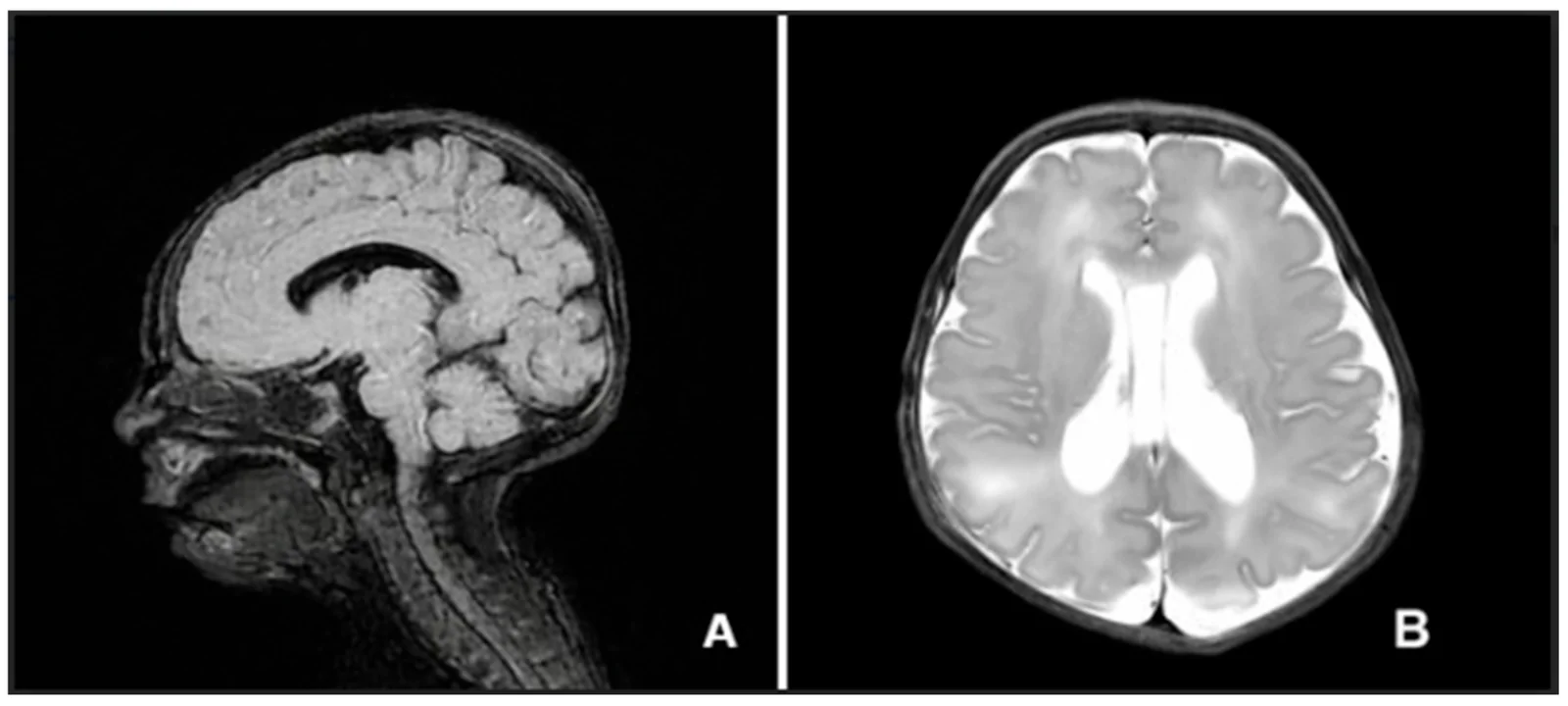
Brain magnetic resonance imaging (MRI). (**A**) Sagittal T1-weighted image demonstrating a subependymal cystic lesion within the left caudothalamic notch. (**B**) Axial T2-weighted image demonstrating bilateral ventriculomegaly with left-sided predominance, colpocephaly, and a cavum vergae variant.

**Table 1:** Endocrinologic findings in the patient with 49,XXXXY syndrome on postnatal day 25.

Investigation	Result	Reference Range
FSH	6.18	<3.3 mIU/mL
LH	8.45	<7 mIU/mL
DHEA-S	634	1.67–861 µg/dL
Androstenedione	0.210	0.180–0.80 ng/mL
17-OHP	0.719	0.030–2 ng/mL
Testosterone	70.7	7–263 ng/dL
Estradiol	27.7	<13 ng/L

FSH, follicle-stimulating hormone; LH, luteinising hormone; DHEA-S, dehydroepiandrosterone sulfate; 17-OHP, 17-hydroxyprogesterone.

**Table 2:** Comparison of previously reported neonatal or early-diagnosed cases of 49,XXXXY syndrome with the present case.

Study	Diagnosis Age	Main Findings	Neuroimaging	Distinctive Feature
Hayek et al. (1971) [7]	Neonatal	Hypotonia, low birth weight	Not reported	Floppy infant presentation
Ng et al. (2007) [8]	Neonatal	Ambiguous genitalia	Not reported	Genital anomalies
Dissanayake et al. (2010) [9]	Birth	Facial dysmorphism, micropenis	Not reported	Cardiac/genital anomalies
Patacchiola et al. (2012) [4]	Neonatal	Clubfoot, micropenis	Ventriculomegaly	Early diagnosis with genital malformations
Kidszun et al. (2012) [10]	Neonatal	Skeletal abnormalities	Not reported	Skeletal findings
Etemadi et al. (2015) [11]	2 months	IUGR, dysmorphism	Not reported	Multisystem anomalies
Tian et al. (2023) [12]	Newborn	IUGR, multiple congenital malformations	Normal cranial ultrasonography and brain MRI findings	Diagnosis by MLPA/QF-PCR
Present case	Neonatal	Ventriculomegaly, cleft palate	Ventriculomegaly, colpocephaly, cavum vergae	Neuroimaging Prompting early genetic evaluation

IUGR, intrauterine growth restriction; MRI, magnetic resonance imaging; MLPA, multiplex ligation-dependent probe amplification; QF-PCR, quantitative fluorescent polymerase chain reaction.

## Data Availability

The data presented in this study are available on request from the corresponding author. The data are not publicly available due to privacy and ethical reasons.

## Abbreviations

IUGR	Intrauterine Growth Restriction
ASD	Atrial Septal Defect
VSD	Ventricular Septal Defect
PDA	Patent Ductus Arteriosus
BAEP	Brainstem Auditory Evoked Potentials
MRI	Magnetic Resonance Imaging
CSF	Cerebrospinal Fluid
FSH	Follicle-stimulating Hormone
LH	Luteinising Hormone
